# Resection of positive tissue on methionine‐PET is associated with improved survival in glioblastomas

**DOI:** 10.1002/brb3.3291

**Published:** 2023-10-16

**Authors:** Kazufumi Ohmura, Takashi Daimon, Yuka Ikegame, Hirohito Yano, Kazutoshi Yokoyama, Morio Kumagai, Jun Shinoda, Toru Iwama

**Affiliations:** ^1^ Chubu Medical Center for Prolonged Traumatic Brain Dysfunction Minokamo Gifu Japan; ^2^ Department of Neurosurgery Gifu University Graduate School of Medicine Gifu Japan; ^3^ Department of Biostatistics Hyogo College of Medicine Nishinomiya Hyogo Japan; ^4^ Chubu Neurorehabilitation Hospital Minokamo Gifu Japan; ^5^ Department of Clinical Brain Sciences Gifu University Graduate School of Medicine Minokamo Gifu Japan; ^6^ Department of Neurosurgery Chubu International Medical Center Minokamo Gifu Japan

**Keywords:** ^11^C‐methionine, extent of resection, glioblastoma, positron emission tomography, survival

## Abstract

**Background and purpose:**

The volume of excised tumor in contrast‐enhanced areas evaluated via magnetic resonance imaging is known to have a strong influence on the survival of patients with glioblastoma (GBM). In this study, we investigated the effect of tumor resection on the survival of patients with GBM in the ^11^C‐methionine (MET) accumulation area using MET‐positron emission tomography (MET‐PET).

**Methods:**

A total of 26 patients (median age, 69 years; 15 males) who had undergone tumor resection and MET‐PET before and after surgery, after being newly diagnosed with GBM, were included in the study. MET‐PET before and after tumor resection were compared. The association between the decrease in the maximum standardized uptake value (SUV) of the tumor divided by the normal cortical mean SUV (%; ΔT/N), the MET extent of resection (MET‐EOR) from the % reduction in the MET accumulation area (%), and residual MET accumulation area (in cm^3^; MET‐residual tumor volume [RTV]), as well as the survival time of patients with GBM, were evaluated via univariate analysis.

**Results:**

ΔT/N were positively associated with survival (hazard ratio [HR], 0.98 [95% confidence interval (CI), 0.97–0.99], *p* = .02). MET‐RTV revealed a negative association with survival (HR, 1.02 [95% CI, 1.01–1.04], *p* = .04). Additionally, MET‐EOR showed a strong trend with survival (HR, 0.99 [95% CI, 0.97–1.01], *p* = .06).

**Conclusions:**

Surgical resection of MET‐accumulated areas in GBM significantly prolongs the survival of patients with GBM. However, a prospective large‐scale multicenter study is needed to confirm our findings.

## Graphical abstract

We aimed to investigate the effect of tumor resection in the ^11^C‐methionine (MET) accumulation area evaluated via MET‐positron emission tomography on the survival of patients with glioblastoma (GBM). Surgical resection of MET‐positive areas in GBMs significantly prolongs the survival of patients with GBM.

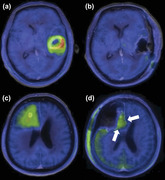

## 1 INTRODUCTION

Glioblastoma (GBM) is the most common intracranial malignancy in adults and known to have a poor prognosis (Stupp et al., 2005). The standard surgical treatment for GBM is tumor resection, with maximum resection of the contrast‐enhanced area of the tumor as assessed via magnetic resonance imaging (MRI). This treatment has been shown to improve the survival rate (Grabowski et al., 2014; Lacroix et al., 2001; Lopez‐Rivera et al., 2021; Sanai et al., 2011). The intraoperative use of 5‐aminolevulinic acid (5‐ALA; Christodoulides & Lavrador, 2021), a neuro‐navigation system based on MRI images of the tumor, and awake surgery are common techniques employed to achieve maximum resection while preserving normal functional tissue (Ius et al., 2023; Youngblood et al., 2021). Recently, the usefulness of fluorescence imaging during GBM surgery has been reported (Cao et al., 2022; Li et al., 2018; Shen et al., 2021; Shi et al., 2022). However, GBMs have a high invasive potential and have been shown to invade more widely than the contrast‐enhanced area evaluated via MRI, making it difficult to determine the resection boundary within the normal brain (Inoue et al., 2021; Miwa, 2004; Pirotte et al., 2006).

^11^C‐methionine‐positron emission tomography (MET‐PET) is a useful tool in neuro‐oncology (Singhal et al., 2008). MET uptake in brain tumors is related to their tissue characteristics, especially their biological activity, any disruption of the blood–brain barrier (BBB), and the volume of blood flow within its borders. It is used in the clinical setting in patients with GBM for diagnostic imaging (Hotta et al., 2019), treatment planning (Pessina et al., 2021), and treatment effect evaluation (Wang et al., 2018). In addition, MET‐PET can determine the boundary between the intracranial tumor and the normal brain more accurately than computed tomography (CT) or MRI (Inoue et al., 2021). However, the use of MET requires a cyclotron, thus making MET‐PET available only to a limited number of facilities.

The impact of MRI‐guided resection of GBM on patient survival has been examined for a long time. Previous reports (Grabowski et al., 2014; Lacroix et al., 2001; Lopez‐Rivera et al., 2021; Sanai et al., 2011) have shown that the extent of resection (EOR) and residual tumor volume (RTV), obtained by comparing MRI scans before and after resection for GBM, are associated with patient prognosis. However, it remains unclear whether the EOR and RTV obtained by comparing MET‐PET before and after surgery are associated with the prognosis of patients with GBM. Moreover, the efficacy of tumor resection in areas with high MET accumulation remains unclear. Since MET‐PET can visualize tumor infiltration more extensively than contrast‐enhanced MRI, surgical resection of the MET accumulation area leads to maximal resection of the tumor (Inoue et al., 2021; Miwa, 2004; Pirotte et al., 2006). Accordingly, we aimed to clarify the effect of surgical resection in the MET accumulation area on the survival of patients with GBM in this retrospective, single‐center study.

## 2 METHODS

### 2.1 Study population

From June 1, 2015, to August 30, 2021, 27 patients with GBM underwent surgical resection, as well as MET‐PET before and after surgery, at Chubu International Medical Center. One patient could not be followed up; thus, the remaining 26 patients were included in the study. Overall survival was defined as the time from surgery to death from any cause. Consent was obtained from all patients. This study was approved by the ethics committee of Chubu Neurorehabilitation Hospital (Number :2022‐10; approval date: October 21, 2022).

### 2.2 Surgery

In all cases, the tumors were resected under a microscope. The surgery was performed using a neuronavigation system. An MET‐PET and MRI co‐registered image, as described 0.6 PET, was used as the baseline for intraoperative navigation. Resection of the tumor, including the area of MET accumulation, was performed. Motor‐evoked potentials were used to maximize tumor removal. No patients underwent intraoperative MRI/CT and awake surgery.

### 2.3 Pathological diagnosis

The diagnosis of all cases was made by pathology and classified according to the 2016 WHO classification (Louis et al., 2016). Isocitrate dehydrogenase (IDH) mutations were determined by pathological diagnosis using anti‐IDH1 (R132) and anti‐IDH2 (R172).

### 2.4 Radiation chemotherapy

After completing the postoperative MET‐PET evaluation, patients were started on concomitant temozolomide (TMZ) at a dose of 75 mg/m^2^ daily during radiotherapy (Stupp et al., 2005). Thereafter, patients received adjuvant TMZ at a dose of 150−200 mg/m^2^ for 5 days in a monthly cycle, followed by 23 days of rest. Adjuvant TMZ was continued until it became no longer viable.

For radiotherapy, the computed tomography, MRI, and MET‐PET datasets were fused using the Pinnacle system (Milpitas). The first gross tumor volume (GTV‐1) was defined as the area with high MET uptake. For high MET uptake, the tumor margin was defined as the area demonstrating a threshold index of 2.0 for the standardized uptake value (SUV) of the tumor, compared with the corresponding area contralateral normal frontal cortex. The second GTV (GTV‐2) was defined as the area with moderate MET uptake, where an index of 1.3 was considered the threshold for defining the tumor margin. Furthermore, with reference to the MRI findings, the first planning target volume was added to GTV‐1 as an additional 5−7 mm margin, and the second planning target volume was obtained by adding the same margin to GTV‐2. A simultaneously integrated boost with intensity‐modulated radiotherapy was performed using a Helical TomoTherapy Hi‐Art System (TomoTherapy Inc.) in eight fractions, with a dose of 68 Gy (biologically effective dose of 126 Gy) targeted to GTV‐1 and a dose of 56 Gy (biologically effective dose of 60 Gy) targeted to the first planning target volume. These doses were prescribed using the 95% isodose line that covered the GTV‐1. The planned irradiation dose for at‐risk organs (eye, optic nerve, and brain stem) was set at <5 Gy (Kawasaki et al., 2019).

### 2.5 MRI

Transaxial T1‐weighted images (repetition time, 2200 ms; echo time, 9.5 ms; number of excitation, 1; inversion recovery delay, 950 ms; field of view, 23 × 23 cm; matrix size, 512 × 510) and T2‐weighted inversion recovery images (repetition time, 3500 ms; echo time, 80 ms; excitations, one; field of view, 230 × 230 mm; and matrix size, 512 × 510) were obtained using a 3T MRI machine (Achieva 3.0T TX QD system: Philips). The slice thickness was 5 mm and the slice gap was 1 mm. A 0.1‐mmol/kg gadolinium‐based contrast agent (Gadobutrol, Gadovist, Bayer HealthCare) was used as the contrast sequence.

### 2.6 PET

PET scans were performed using an Eminence STARGATE (Shimadzu Corp.), with a three‐dimensional acquisition mechanism. A total of 35 axes were acquired at 2.65‐mm intervals, with a spatial resolution of 4.8 mm. Three laser beams from the patients’ facial scans were used to adjust the axial slices parallel to the luminal lines. Seven minutes after the transmission scan, MET was administered intravenously. The MET dose was 3.5 MBq/kg, and contrast‐enhanced MRI was performed on the same day after completion of the PET scan. At a median of 12 days (range, 3−26 days) after surgery, postoperative PET and MRI were performed on the same day; PET images were fused with MRI images for anatomical evaluation, according to the method of Kapouleas et al. (1991).

### 2.7 Image evaluation

MET accumulation in the region of interest (ROI) was analyzed from the determined SUV. The ROI was manually drawn, and the maximum SUV of the tumor divided by normal cortical mean SUV (T/N ratio) was calculated. Similar to previous reports (Galldiks et al., 2012; Kracht et al., 2004), areas with T/N ratios greater than 1.3 were measured as the MET volume of accumulation. If no MET volume accumulation was observed postoperatively, ROIs were set in the region where MET accumulation was observed preoperatively. The preoperative tumor volume (PTV) and postoperative RTV were measured in each patient in three areas: the MET‐accumulated area, the gadolinium contrast‐enhancing (Gd) area, and the T2 high‐signal area. All tumors were enhanced by gadolinium. In each axial MRI slice, the PTV and RTV were bordered manually, and each slice was summed and measured. Reduction in the T/N ratio (ΔT/N) and EOR were calculated as follows:

$$\begin{array}{ccc} \Delta T/N & = & \left(\mathrm{preoperative}\;T/N\;\mathrm{ratio}-\mathrm{postoperative}\;T/N\;\mathrm{ratio}\right)/\\ & & \mathrm{preoperative}\;T/N\;\mathrm{ratio}\times 100\;\mathrm{and}\;\mathrm{EOR}=\left(\mathrm{PTV}-\mathrm{RTV}\right)/\\ & & \mathrm{PTV}\times 100. \end{array}$$

**FIGURE 1:**
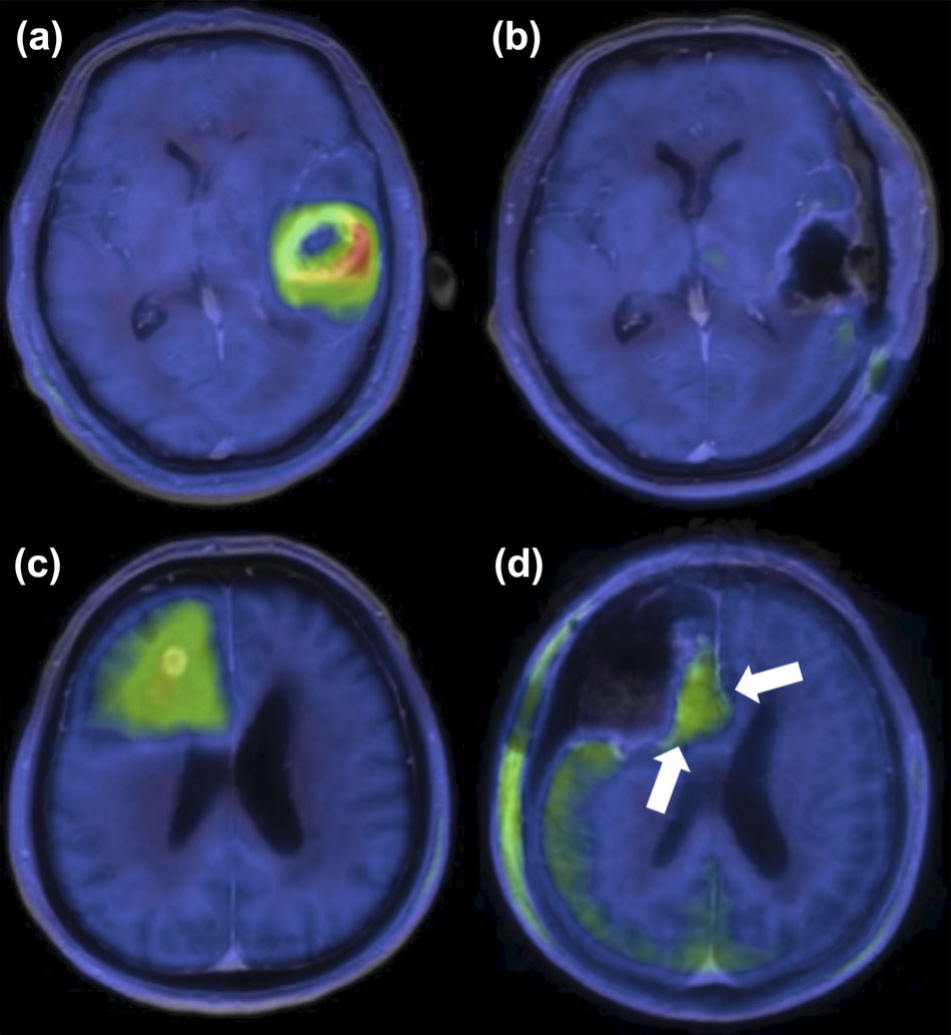
Illustration of typical cases of glioblastoma with preoperative and postoperative ^11^C‐methionine‐positron emission tomography (MET‐PET). (a–d) Gadolinium‐enhanced T1‐weighed images co‐registered with MET‐PET. (a and b) A case of 63‐year‐old woman. (a) Preoperative MET‐PET showed high MET accumulation in the lesion. (b) Postoperative MET‐PET showed no residual high MET uptake. (c and d) A case of a 78‐year‐old man. (c) Preoperative MET‐PET. MET accumulation was observed beyond the gadolinium‐enhanced area. (d) Postoperative MET‐PET. Arrows indicated residual MET accumulation measured at a threshold of 1.3 times the normal cortical mean standardized uptake value.

For postoperative image evaluation, a T1 subtraction map was created by excluding the volume with a high signal on postoperative T1‐weighed images from the postoperative Gd volume to circumvent the influence of surgery as demonstrated in previous reports (Ellingson et al., 2018; Grabowski et al., 2014; Lacroix et al., 2001; Sanai et al., 2011). Postoperative T2‐weighed images were measured in the T2 high‐signal region, excluding the resection cavity. Analyses were conducted by the corresponding author, who was blinded to the survival rates and EOR data according to other modalities. Dr. View/Linux image analysis software (Infocom Corp.) was used to measure image fusion, SUV, and tumor volume. Figure 1 shows a typical case of GBM with preoperative and postoperative MET‐PET scans.

### 2.8 Statistics

Continuous and categorical variables are presented as medians with ranges and frequencies with proportions, respectively. Overall survival curves were estimated using the Kaplan–Meier method and compared using the log‐rank test. Owing to the small number of events (only 17 of 26 patients), no multivariable analysis was performed to adjust for the effects of the prognostic factors. Instead, univariate analysis was performed using the Cox proportional hazards model to evaluate the association of each image evaluation variable with overall survival. Furthermore, thresholds for MET‐EOR were explored using the Cox proportional hazards model to associate them with overall survival (Grabowski et al., 2014; Lacroix et al., 2001). The results were summarized as hazard ratios (HRs), 95% confidence intervals (CIs), and *p*‐values. All *p*‐values were two‐sided, and a *p*  <  .05 was considered to be statistically significant. All data analyses were conducted using JMP 14.2.

## 3 RESULTS

### 3.1 Patient characteristics

All 26 patients underwent a one‐time surgical resection followed by chemotherapy and radiotherapy as described above. At the time of analysis, nine patients were confirmed to be alive. Table 1 presents the general patient characteristics data. The average age of the 26 enrolled patients was 69 (range: 48−83 years old). Of the patients, 15 (57.7%) were male, with a median age of 68 years (range: 48−82 years), and 11 (42.3%) were female, with a median age of 70 years (range: 51−83 years). All diagnoses were made by pathology: IDH wild‐type in 15 (57.7%), not otherwise specified in 11 (42.3%), and mutant type in 0. The median preoperative Karnofsky Performance Scale (KPS) score was 80 (range: 60−90), and the median recursive partitioning analysis was 5 (range: 4−6). The survival time was 20.1 (range: 1.0−68.8) months. At the time of analysis, the median follow‐up time was 18.2 (range: 3.1−68.8) months.

**TABLE 1:** Patient population.

Variable	Value
Patient	26 cases
Sex number (%)	Male 15 (57.7%), female 11(42.3%)
Age, years	69 (48−83)
Isocitrate dehydrogenase wild‐type, number (%)	15 cases (57.7%)
Not otherwise specified, number (%)	11 cases (42.3%)
Karnofsky performance status	80 (60−90)
Recursive partitioning analysis	5 (4–6)
Median survival (95% confidence interval)	20.1 months (13.4−35.8)

*Note*: All data represent median (range) unless otherwise indicated.

### 3.2 Univariate analysis for overall survival

The imaging data and HR of each dataset are listed in Table 2. The association with overall survival was statistically significant for the ΔT/N ratio (*p* = .02; HR, 0.98 [95% CI 0.97−0.99]) and MET‐RTV (*p* = .04; HR, 1.02 [95% CI 1.01−1.04]). There seemed to be some association for MET‐EOR (*p* = .06; HR, 0.99 [95% CI 0.97−1.01]), preoperative T/N ratio (*p* = .06; HR, 0.61 [95% CI 0.32−1.02]), and Gd‐RTV (*p* = .052; HR, 1.03 [95% CI 0.99−1.05]), although these were not statistically significant. Gd‐PTV (*p* = .77; HR, 1.01 [95% CI 0.98−1.02]) and Gd‐EOR (*p* = .11; HR, 0.99 [95% CI 0.98−1.01]) were not statistically significant for survival.

**TABLE 2:** Imaging data and analysis of hazard ratio (HR) for survival.

	Median (range)	HR (95% CI)	*p*‐value
Preoperative imaging			
T/N ratio	4.0 (2.1−7.0)	0.61 (0.32−1.02)	.06
MET‐PTV	37.3 (10.1−127.5)	1.02 (0.99−1.04)	.07
Gd‐PTV	40.2 (1.7−96.2)	1.01 (0.98−1‐02)	.77
T2‐PTV	127.0 (7.2−250.0)	0.99 (0.98−1.01)	.16
Postoperative imaging			
T/N ratio	2.3 (0.9−5.5)	1.28 (0.89−1.81)	.18
MET‐RTV	7.2 (0−114.1)	1.02 (1.01−1.04)	.04^*^
Gd‐RTV	3.7 (0−92.4)	1.03 (0.99−1.05)	.052
T2‐RTV	47.6 (0.5−109.6)	0.99 (0.97−1.01)	.25
Comparison between before and after surgery			
ΔT/N%	31.2 (−33.0−80.7)	0.98 (0.97−0.99)	.02^*^
MET‐EOR%	82.7 (6.4−100)	0.99 (0.97−1.01)	.06
Gd‐EOR%	84.5 (−25.6−100)	0.99 (0.98−1.01)	.11
T2‐EOR%	64.0 (8.5−96.5)	1.01 (0.99−1.03)	.42

*Note*: ΔT/N = (preoperative T/N ratio − postoperative T/N ratio)/preoperative T/N ratio × 100; EOR, extent of resection, (PTV—RTV)/PTV × 100.

Abbreviations: CI, confidence interval; Gd, the gadolinium contrast enhancing; MET = ^11^C‐methionine; PTV, preoperative tumor volume (cm; Sanai et al., 2011); RTV, residual tumor volume (cm; Sanai et al., 2011); T/N = the maximum standardized uptake value (SUV)/normal cortical mean SUV.

* indicates statistical significance.

### 3.3 Comparison of patient survival according to various MET‐EOR

MET‐EOR was analyzed in an exploratory manner (using Cox proportional HR) by varying the value by 1% for further analysis (Table 3), similar to previous reports on Gd‐EOR (Grabowski et al., 2014; Lacroix et al., 2001). Statistical significance was achieved with resections of 84% or more.

**TABLE 3:** Comparison of patient survival according to various ^11^C‐methionine‐extent of resection.

Cutoff point (%)	Below No. of cases	Cutoff point Median survival in months (95% CI)	Above No. of cases	Cutoff point Median survival in months (95% CI)	HR (95% CI) Below versus above	*p*‐value (Cox hazard)
>89	15	15.5 (5.8−28.3)	11	34.1 (9.0–∞)	0.33 (0.10−0.93)	.04^*^
>88−84	14	15.5 (5.8−19.2)	12	35.8 (12.0–∞)	0.26 (0.08−0.76)	.01^*^
>83	13	15.5 (5.8−28.3)	13	28.5 (9.0−47.0)	0.35 (0.11−1.00)	.05
>82	13	15.5 (5.8−28.3)	13	28.5 (9.0−47.0)	0.35 (0.11−1.00)	.05
>81	12	15.5 (5.8−28.3)	14	28.5 (9.0−47.0)	0.40 (0.14−1.14)	.09

*Note*: The number of cases indicates all patients with MET‐EOR above or below the cutoff value.

Abbreviations: CI, confidence interval; No, number.

* indicates statistical significance.

### 3.4 Overall survival for MET‐EOR > 84% and MET‐EOR ≤ 84

**FIGURE 2:**
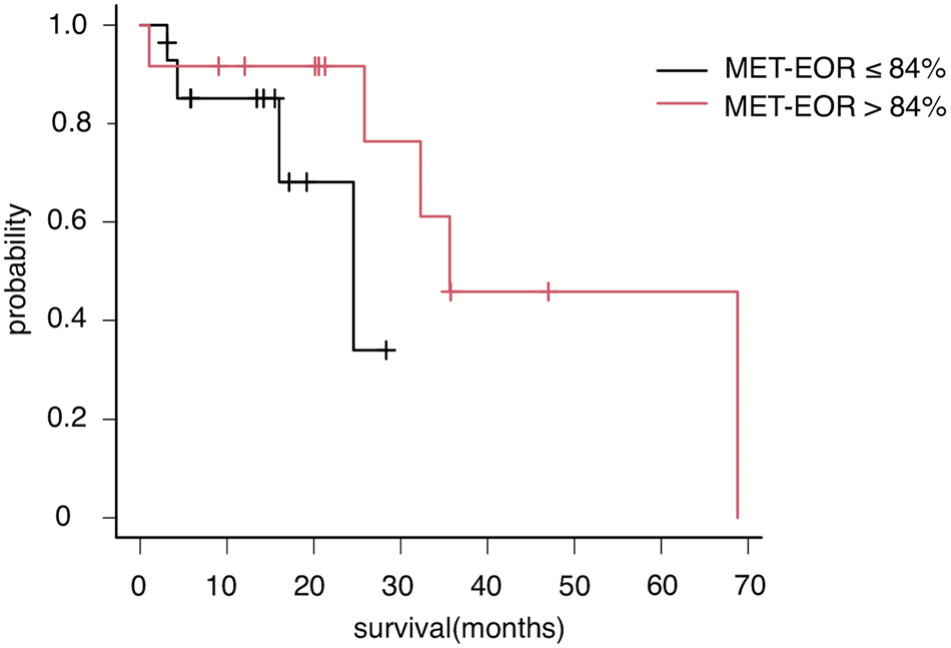
Overall survival curves for MET‐extent of resection (EOR) > 84% group and MET‐EOR ≤ 84% group. The median survival was 35.8 months (95% confidence interval [CI] 12.0–∞) in the MET‐EOR > 84% group (12 cases, red line) and 15.5 months (95% CI 5.8−19.2) in the MET‐EOR ≤ 84% group (14 cases, black line). The estimated survival was significantly different among the two groups (*p* = .01 by log‐rank test).

The overall survival curves for survival between the MET‐EOR > 84% group (*n* = 12) and the MET‐EOR ≤ 84% group (*n* = 14) were obtained. A significant difference was observed in survival time between the two groups (*p* = .01, log‐rank test; Figure 2). Table 4 shows a comparison of survival time between MET‐EOR > 84% and Gd‐EOR > 70%, a threshold reported by Chaichana et al. (2014).

**TABLE 4:** Comparison of different resection targets for survival.

Resection outcome	MET‐EOR > 84%	Gd‐EOR > 70 %^*^
% 1‐year OS (95% CI)	90.9 (73.9−100)	86.7 (69.5−100)
% 1.5‐year OS (95% CI)	81.8 (55.5−100)	66.7 (42.8−90.5)
% 2‐year OS (95% CI)	54.6(25.1−84.0)	46.7 (21.4−71.9)

Abbreviations: CI, confidence interval; EOR = extent of resection, (preoperative tumor volume − residual tumor volume)/preoperative tumor volume × 100; Gd, the gadolinium contrast enhancing; OS = overall survival; MET = ^11^C‐methionine.

* The threshold reported by Chaichana et al.^2014^,.&nbsp;.

## 4 DISCUSSION

We studied the impact of the surgical resection of areas with MET accumulation on the survival of patients with GBM. Surgical resection of the area of MET accumulation significantly affected patient survival positively.

MET‐PET was widely used in neuro‐oncology (Singhal et al., 2008). For GBM, it was useful for diagnostic imaging (Kato et al., 2008), treatment planning (Pessina et al., 2021; Pirotte et al., 2006), and determination of the efficacy of chemotherapy (Hirono et al., 2019; Miller et al., 2020; Wang et al., 2018). Moreover, GBM was known to be a malignant disease with a poor prognosis, with a reported survival time of about one and a half years (Stupp et al., 2005). The standard treatment for GBM was surgical resection combined with TMZ and radiotherapy (Stupp et al., 2005). Additionally, GBM was associated with a high rate of local recurrence, which was associated with a poor prognosis. Grabowski et al. (2014) reported that Gd‐EOR and Gd‐RTV were predictors of survival after GBM resection, with Gd‐RTV being the more important predictor. Ellingson et al. (2018) reported that Gd‐RTV is a predictor of survival, regardless of age, post‐op treatment, or O6‐methylguanine‐DNA methyltransferase (MGMT) methylation. In summary, it is clear that extended resection of the GBM to the greatest extent possible is important for prolonging survival. It has also been reported that GBM resection based on the maximum tumor resection defined by the conventional MRI‐based Gd area (Grabowski et al., 2014; Lacroix et al., 2001; Lopez‐Rivera et al., 2021; Sanai et al., 2011) or fluid‐attenuated inversion recovery high‐signal area led to prolonged survival (Certo et al., 2021). Some authors have also used 5‐ALA intraoperatively and reported the efficacy of tumor resection (Christodoulides & Lavrador, 2021). However, few studies have reported the association between tumor resection focusing on MET accumulation areas and the survival of patients with GBM.

A report found that resection of MET‐accumulated areas beyond the Gd area improved survival in GBM patients (*p* = .03, log‐rank test; Hirono et al., 2021). However, the EOR and postoperative MET accumulation were unclear. Pirotte et al. (2009) reported that the absence of postoperative MET accumulation on imaging was associated with prolonged survival (HR, 0.532; *p* = .0001) in 31 GBM patients. Similar results were obtained, indicating that the absence postoperative MET accumulation was associated with survival. Additionally, we investigated postoperative MET‐PET findings, using ΔT/N ratio, MET‐EOR, and MET‐RTV, and their association with survival, providing new predictors for GBM resection survival.

A high T/N ratio indicates increased amino acid metabolism, blood flow, rich vascular bed, and disruption of the BBB in the lesion, showing the lesion's high biological activity (Singhal et al., 2008). It is known that MET accumulation was higher in malignant brain tumors (Kato et al., 2008). Wang et al. (2018) employed MET‐PET to evaluate GBM response to radio‐chemotherapy and found a 40% reduction in the T/N ratio before and after treatment. Moreover, changes in the SUV of MET and MET accumulation volume correlated with MGMT methylation before and after radiotherapy, suggesting that MET‐PET is useful for assessing GBM response to radio‐chemotherapy. No study has quantitatively evaluated the changes in MET accumulation before and after surgery in patients with GBM. In our study, the median ΔT/N was 31.2%, and the rates of decrease in the T/N ratio after surgery were predictors of survival. Surgical resection of MET‐accumulated areas with high activity that cannot be assessed using Gd‐MRI is important for prolonging survival. Thus, MET‐PET may be superior to Gd‐MRI for assessing residual lesion activity after surgery.

It has been reported that MET accumulation is more extensive than that found in Gd areas, indicating that tumor cells are more likely to infiltrate normal tissues (Inoue et al., 2021; Miwa, 2004; Pirotte et al., 2006). GBM was found to be more invasive when MET‐accumulated areas were beyond the Gd area, and recurrence in these areas confirmed the importance of resection of MET‐accumulated areas (Miwa, 2004). In general, malignant gliomas are known to recur locally when a residual tumor is found, and maximum tumor resection is required to be performed. Previous studies have reported that the Gd‐RTV is an important predictor of survival on postoperative imaging. Grabowski et al. (2014) and Chaichana et al. (2014) demonstrated that a Gd‐RTV greater than 2 or 5 cm (Sanai et al., 2011) was associated with a difference in survival. However, Molinaro et al. (2020) showed that in patients with GBM below the age of 65 years with IDH wild‐type, in addition to maximum resection of the Gd area, resection of the non‐Gd area of the lesion which was <5.4 mL associated with prolonged survival (overall median survival, 37.3 vs. 16.5 months) was noted, which was comparable to the prognosis of the IDH mutant type. Other reports indicated that lobectomy in the tumor region, rather than resection of the contrast‐enhanced lesion, prolongs survival (Shah et al., 2020). In terms of MET‐PET, previous reports had demonstrated that postoperative accumulation of MET could make a difference in the survival of patients with GBM (Hirono et al., 2021). Several reports supported that resection beyond the Gd area of GBM led to prolonged survival. In our study, Gd‐RTV exhibited a strong but not statistically significant association with the survival of patients with GBM (HR, 1.03 [95% CI 0.99−1.05], *p* = .052). However, MET‐RTV was shown to be a prognostic factor for survival (HR, 1.02 [1.01−1.04], *p* = .04). It is therefore important to focus on residual MET accumulation, in addition to the Gd area, in postoperative patients with GBM.

Nevertheless, MET‐EOR, which measures the percentage reduction in the volume of MET accumulation, exhibited a strong significant trend with survival but did not reach statistical significance (HR, 0.99 [95% CI 0.97−1.01], *p* = .06). Miwa (2004) reported that MET accumulation within 5 mm of the Gd area in GBM was only 80.3% but expanded to 99.8% when the area was 3 cm. Tumor resection was effective in GBM but did not prolong survival with reduced neurological function (Karsy et al., 2018). Furthermore, the prognosis was poor for elderly patients with surgical complications (Voisin et al., 2021). In a report by Molinaro et al. (2020), patients with GBM < 65 years of age underwent maximum resection of Gd areas as well as resection of non‐Gd areas. Conversely, patients aged >65 years underwent resection of only the Gd areas. In our study, most patients were elderly (median age, 69 years), and we did not examine postoperative neurological function or complications; however, it is possible that resection of the MET‐accumulated area did not contribute to prolonged survival owing to decreased neurological function. Previous validation of resections in Gd areas reported a survival difference ranging from a minimum of 70% to 100% (Chaichana et al., 2014; Ellingson et al., 2018; Grabowski et al., 2014; Lacroix et al., 2001; Sanai et al., 2011). A report that compared MET accumulation before and after surgery demonstrated prolonged survival in the group with no postoperative accumulation (Hirono et al., 2021), but the validation of the EORs in the MET accumulation area was unclear. We examined the EOR by varying the MET reduced volume in 1% increments, similar to other reports searching for the EOR in Gd areas (Grabowski et al., 2014; Lacroix et al., 2001), to determine the EOR that would be significant for survival. MET‐EOR greater than 84% was significant for survival (HR, 0.26 [0.08−0.76], *p* = .01). These results indicate that the resection of the MET‐accumulated area above a certain level is effective. However, further investigations are needed to determine the MET‐EOR threshold required to prolong survival in patients with GBM, and a larger prospective study is necessary to confirm our results.

Our study found that maximum resection of GBM in areas with MET accumulation improved survival, along with the resection of tumor areas assessed by conventional Gd‐MRI. Furthermore, resecting MET‐accumulated areas with a high T/N ratio, which indicates high tumor activity, was crucial, in addition to the Gd area and invasion extent. This may help in planning GBM resection near the eloquent area. Targeting more active areas with a higher T/N ratio during surgical resection, without causing neurological dysfunction, can reduce the T/N ratio and potentially prolong survival (Certo et al., 2021). In addition, for GBM in the non‐eloquent area, maximum resection of both the MET‐accumulated area and the Gd area can lead to prolonged survival. However, it is important to note that aggressive resection carries the risk of worsening neurological deficits or causing complications, ultimately leading to an unfavorable prognosis (Karsy et al., 2018; Voisin et al., 2021).

As the first investigation in this direction, this study has the following limitations. Only 26 patients were enrolled because of limited insurance coverage and facilities. We only conducted univariate Cox analysis because of the small sample size and could not adjust for age, KPS, or genetic factors. In addition, MGMT methylation or genetic mutations (Ferguson et al., 2021) were not evaluated in all cases. The MGMT methylation status has a profound impact on the survival of patients with GBM, separate from EOR. The association between resection of the MET uptake area and outcomes should be investigated with consideration of MGMT mutations. Postoperative MRI was conducted at a later stage than recommended in the current guidelines (Grabowski et al., 2014; Ius et al., 2023), which may have hindered our ability to accurately assess the postoperative Gd area (Garcia‐Ruiz et al., 2021). On the other hand, the threshold of Gd‐EOR > 70% we used for comparison is typically applied to postoperative MRIs performed at shorter intervals. These differences in intervals may have affected the results. Therefore, studies that adhere to the optimal timing of postoperative MRI are required. Only a few patients had known postoperative KPS scores. Whether KPS declines due to resection of the MET uptake area should be explored. Tumor treating fields therapy was not evaluated. Although tumor treating fields therapy was not evaluated in this study, its use has been shown to have a positive influence on the outcome of patients with GBM (Stupp et al., 2017). There were nine out of 26 survivors, which may have influenced the outcome of the survival analysis. As previously reported by Sanai et al. (2011), there was a problem with the statistical strategy used to determine the EOR in MET‐accumulated areas. Although dichotomizing continuous predictors to discuss some cut points related to the outcome is frequently practiced in clinical medicine, it has been reported that it generates serious biases (Royston et al., 2006). However, there have been no reports exploring the EOR in MET‐accumulated areas following GBM surgery as it relates to prolonged survival, and we followed previous important reports (Grabowski et al., 2014; Lacroix et al., 2001) that examined the EOR in Gd areas and residual Gd volume after GBM surgery. It was unclear whether the high T/N areas corresponded to the Gd areas, and if they did, to what extent the areas corresponded to each other. Given that MET accumulation is more extensive than Gd areas, studies focusing on the resection of MET‐accumulated, non‐Gd areas are needed (Inoue et al., 2021; Miwa, 2004, Pirotte et al., 2006). Recent MRI‐based studies suggest that the resection of a non‐Gd area beyond the Gd area may be beneficial for survival (Certo et al., 2021; Molinaro et al., 2020). Consequently, it is also necessary to evaluate the impact of different RTV or EOR thresholds for non‐MET‐accumulated areas on survival. It would be worthwhile to further analyze both Gd‐MRI and MET‐PET findings in this study. This provides an opportunity to investigate the overlap more thoroughly and may contribute to a more comprehensive understanding of MET‐PET in GBMs.

In conclusion, our study examined how the surgical resection of areas of MET accumulation affects the survival of patients with GBM. Surgical resection of the area of MET accumulation positively affected patient survival. Whether more extensive surgical resection of MET‐accumulated areas beyond the corresponding Gd areas leads to prolonged patient survival needs to be confirmed in a subsequent prospective, large‐scale, multicenter study.

## AUTHOR CONTRIBUTIONS

**Kazufumi Ohmura**: Conceptualization; investigation; methodology; formal analysis; project administration; writing—original draft. **Takashi Daimon**: Formal analysis; writing—review and editing. **Yuka Ikegame**: Formal analysis; investigation; writing—review and editing. **Hirohito Yano**: Writing—review and editing. **Kazutoshi Yokoyama**: Resources; writing—review and editing. **Morio Kumagai**: Investigation; writing—review and editing. **Jun Shinoda**: Writing—review and editing. **Toru Iwama**: Resources; Supervision; writing—review and editing. All authors read and approved the final manuscript.

## CONFLICT OF INTEREST STATEMENT

The other authors state that they have no financial competing interests.

### FUNDING STATEMENT

The authors received no specific funding for this work.

### PEER REVIEW

The peer review history for this article is available at https://publons.com/publon/10.1002/brb3.3291

## Data Availability

The datasets generated or analyzed during the current study are available from the corresponding author upon reasonable request.
